# Xanthatin‐13‐(Pyrrolidine‐2‐Carboxylic Acid), a Sesquiterpene Lactone Isolated From Burdock Leaf, Attenuated Aβ_25‐35_ Toxicity and Memory Deficits in a Pharmacological Mouse Model of Alzheimer's Disease

**DOI:** 10.1002/ptr.70294

**Published:** 2026-03-07

**Authors:** Charlyne Barry‐Simonnet, Lucie Crouzier, Tristan Moujellil‐Legagneur, Hamza Chaieb‐Errass, Yanis A. Idres, Karine Ferrare, Marc Rolland, Guillaume Cazals, Patrick Poucheret, Tangui Maurice, Didier Tousch

**Affiliations:** ^1^ MMDN Univ Montpellier, EPHE, INSERM Montpellier France; ^2^ Qualisud Univ Montpellier, CIRAD, Institut Agro, Univ Avignon, Univ La Réunion Montpellier France; ^3^ IEM Univ Montpellier, CNRS, ENSCM Montpellier France; ^4^ PAC Chimie‐Balard Univ Montpellier, CNRS, ENSCM Montpellier France

**Keywords:** anti‐amnesia, drug combination, neuroinflammation, neuroprotection, oxidative stress, sesquiterpene alkaloid

## Abstract

Alzheimer's disease (AD) is a severe form of dementia, which occurrence increases with age and lifestyle conditions. It is characterized by amyloid protein accumulation forming senile plaques, hyperphosphorylated tau protein forming neurofibrillary tangles, neuroinflammation, and oxidative stress, leading to synapse loss and cell death. Pharmacological alternatives to conventional treatments include alkaloids with anti‐inflammatory and antioxidant properties. Sesquiterpene lactones, such as Xanthatin‐13‐(pyrrolidine‐2‐carboxylic acid) (XPc) from burdock leaf, show promise due to their antioxidant activity targeting glucose‐6‐phosphate dehydrogenase. This study evaluated XPc's protective effects in vivo using Aβ_25‐35_‐treated mice, a pharmacological AD model, and explores its synergistic potential with neuroprotective agents like TSPO activators or sigma‐1 receptor agonists. Mice were administered Aβ_25‐35_ peptide (9 nmol ICV) and XPc (0.3–3 mg/kg) daily for 4 days. Behavioral tests assessed memory deficits and anxiety. Post‐sacrifice, brains were analyzed for neuroinflammation and oxidative stress markers. Combination studies involved XPc with the TSPO activator PK11195 or the sigma‐1 receptor agonist PRE‐084, with memory evaluated in two behavioral tests. Combination indices were calculated to assess synergy. XPc (1–3 mg/kg) prevented Aβ_25‐35_‐induced memory impairments and anxiety. It reduced astroglial reaction, blocked microglial activation, and confirmed antioxidant activity by lowering lipid peroxidation and protein nitrosylation. Combinations with PK11195 or PRE‐084 showed synergistic protection in memory tests. XPc is a potent neuroprotective agent against AD‐like toxicity in this murine model, effective alone or in synergistic combinations with other drugs.

**Abbreviations**
AD: Alzheimer's disease
ARIA: amyloid‐related imaging abnormalities
ATF4: (activating transcription factor 4)
Aβ: amyloid‐β peptide
Aβ_25‐35_
: amyloid‐β[25–35] peptide
BACE‐1: β‐site APP cleaving enzyme‐1
BiP: binding immunoglobulin protein
CI: combination indexes
COX: cyclooxygenase
DHFR: dihydrofolate reductase
DS1R: sigma‐1 receptor
EIF2α: eukaryotic initiation factor 2α
ER: endoplasmic reticulum
G6PD: glucose‐6‐phosphate dehydrogenase
GFAp: glial fibrillary acidic protein
GPx: glutathione peroxidase
GSH: glutathione
GSSG: oxidized glutathione
HO‐1: heme oxygenase‐1
HRP: horseradish peroxidase
IBA‐1: allograft inflammatory factor 1
ICV: intracerebroventricular
IDE: insulin‐degrading enzyme
IL‐6: interleukin‐6
IP: intraperitoneal
ITI: intertrial time interval
Keap1: Kelch‐like ECH‐associated protein 1
LPS: lipopolysaccharide
MAM: mitochondria‐associated ER membrane
NADPH: nicotinamide adenine dinucleotide phosphate
NOS: nitric oxide synthase
Nox: NADPH‐oxidase enzyme
Nrf2: nuclear factor‐erythroid‐2‐related factor 2
PBS‐T: phosphate buffer saline‐Tris
PO: *per os* by gavage
PP: percentage of protection
Sc.Aβ: scrambled amyloid peptide
SOD: superoxide dismutase
TDP43: TAR DNA‐binding protein 43
TNFα: tumor necrosis factor α
TSPO: translocator protein
V: vehicle solution
XPc: Xanthatin‐13‐(pyrrolidine‐2‐carboxylic acid)

## 1 Introduction

Alzheimer's disease (AD) is a progressive neurodegenerative disease characterized by devastating cognitive deficits and complete loss of autonomy ultimately leading to dementia and death (Bondi et al. 2017). The economic burden due to AD and related dementia in developed countries is constantly rising and new therapeutical solutions are proposed to manage the disease progression but no curative nor real disease‐modifying treatment is yet available. Symptomatic treatments included three cholinesterase inhibitors and an uncompetitive antagonist of the N‐methyl‐D‐aspartate type of glutamate receptors (Tan et al. 2014) but newly available therapies target the key physiopathological features in AD, the aggregation of pathological proteins. Indeed, the AD brain exhibits characteristic pathological extracellular accumulations of amyloid‐β (Aβ) proteins forming deposits and ultimately senile plaques and intracellular aggregation of hyperphosphorylated tau protein, disorganizing the cellular cytoskeleton and leading to neurofibrillary tangles. The recently developed passive immunization therapies target Aβ species and contribute to lower amyloidopathy but with frequent adverse side‐effects such as amyloid‐related imaging abnormalities (ARIA) (Sperling et al. 2011; Chundu et al. 2025). Neurodegeneration in AD also relies on a massive neuroinflammation and oxidative stress and a widespread neuronal loss (Butterfield and Halliwell 2019). Neuroinflammation has long been considered an unspecific consequence of Aβ and tau accumulations but is now seen as a main driver in AD pathology (Szlufik et al. 2024). Oxidative stress is both a cause and consequence of neuroinflammation, deregulation of cellular anti‐oxidant enzymes and mitochondrial alterations in neurons and glial cells (Götz et al. 1994; Mark et al. 1996; Meng et al. 2025). For instance, (Martins et al. 1986) described that in AD patients, the activities of both 6‐phosphogluconate dehydrogenase and glucose‐6‐phosphate dehydrogenase enzymes were increased, the activity of the latter being almost double the activity of normal controls.

Among plant alkaloids and other naturally occurring organic compounds, numerous present strong anti‐oxidant properties and reduce reactive oxygen species levels or boost anti‐oxidant enzyme activities, such as vitamins, polyphenols, flavonoids, coumarins, terpenoids or sesquiterpene lactones (Zhao 2009; Ullah et al. 2023; Prabha et al. 2025). In particular, they activate the Keap1‐Nrf2‐ARE signaling pathway known to play a crucial role in regulating cellular responses to oxidative stress (Zhao et al. 2025). By neutralizing free radicals and enhancing antioxidant defenses, these natural products may offer a promising approach to counteract AD pathology or even to sustain the protection induced by chemicals acting on other specific pathways. Among them, we previously described Xanthatin‐13‐pyrrolidine‐2‐carboxylic acid (XPc), a sesquiterpene lactone originally isolated by low‐pressure liquid chromatography and LC–MS‐RMN from leaf extracts of burdock ( *Arctium lappa* L.) using a screen of different bitter *Asteraceae* plant fractions to identify anti‐oxidant active components (Idres et al. 2021). XPc protected at micromolar concentrations against the H_2_O_2_‐induced L6 cell death and increased the expression of glucose‐6‐phosphate dehydrogenase (G6PD), but not superoxide dismutase (SOD) or glutathione peroxidase (GPx), among anti‐oxidant enzymes. Molecular docking analyses confirmed a direct interaction between XPc and G6PD (Idres et al. 2021), suggesting a different activation pathway not involving the transcription factor Nrf2, known to activate all three genes. XPc therefore targets a cytosolic enzyme and displays strong anti‐oxidant and anti‐inflammatory potency.

In the present study, we analyzed the protective potency of XPc in a pharmacological mouse model of AD induced by intracerebroventricular administration of oligomerized Aβ_25‐35_ peptide (Maurice et al. 1996). The model is particularly suitable for the initial demonstration of the neuroprotective potential of new investigational drugs as it presents all cellular and biochemical aspects of the AD‐like toxicity including the generation of amyloid‐β species and the hyperphosphorylation of tau protein, but not leading to amyloid plaques and neurofibrillary tangles, respectively, as it remains an acute insult model (for a recent review, see Canet et al. 2023). However, all markers of oxidative stress or neuroinflammation are significantly expressed and the model allows the characterization of drugs with potent anti‐inflammatory and/or anti‐oxidant activities. XPc was administered intraperitoneally during 4 days after the peptide and mice were analyzed after 1 week and over 2 weeks using a battery of behavioral tests assessing different types of memory and anxiety (spontaneous alternation, object recognition, place learning in the water maze, passive avoidance and marble burying). Neuroinflammation was then assessed in the hippocampal by an immunofluorescence analysis of reactive astrocytes and microglia and by the tissue content in tumor necrosis factor α (TNFα) and interleukin‐6. And oxidative stress was also analyzed using a lipid marker (lipid peroxidation) and a protein marker (the level of nitrosylation of proteins).

In a last series of experiments, we examined the potentialities of using XPc in combination therapies with other neuroprotective agents developed in AD and targeting complementary anti‐oxidant and anti‐inflammatory pathways. The translocator protein TSPO is located on the outer mitochondrial membrane and is involved in cholesterol and other steroid transport in the mitochondria (Mokrov et al. 2021). TSPO ligands are not only useful as biomarkers for neuroinflammation and thus developed as bioimaging agents (McNeela et al. 2018; Roveta et al. 2024). The sigma‐1 receptor (S1R) is a ligand‐operated chaperone localized on the endoplasmic reticulum (ER) membrane and particularly enriched in mitochondria‐associated ER membranes (MAMs). Agonists activating S1R are neuroprotective in preclinical studies of neurodegenerative diseases and one agonist, blarcamesine, is currently tested in phase 2b/3 clinical trials (Villard et al. 2011; Macfarlane et al. 2025). Therefore, we combined XPc and the TSPO activator PK11195 or the S1R agonist PRE‐084, and analyzed the effects of the mix on Aβ_25‐35_‐induced memory impairments in the spontaneous alternation and passive avoidance responses, to determine whether the drugs could lead to synergistic effects by affecting different cellular pathways originating from the cytosol, mitochondria, or ER.

## 2 Material and Methods

### 2.1 Animals

A total of 277 male Swiss CD‐1 (RjOrl:SWISS) mice (7–9 weeks old) were obtained from Janvier Labs (Le Genest‐Saint‐Isle, France) and housed in the University of Montpellier animal facility (CECEMA, agreement #D34‐172‐23). Animals were group‐housed (5–10 per cage) with ad libitum access to food and water under controlled conditions (23°C ± 1°C, 40%–60% humidity, 12 h/12 h light/dark cycle). Behavioral experiments were conducted between 09:00 and 16:00 h. All procedures complied with EU Directive 2010/63/EU, ARRIVE guidelines, and were approved by the French National Ethics Committee (APAFIS #48359–202,403,211,416,309). Drug doses were randomized, but experiments were not blinded. Requirements considered to be relevant in recent guidelines for best practice in natural products pharmacological research have been taken into account (Heinrich et al. 2020; Wang et al. 2024).

### 2.2 Compounds and Administration Routes

XPc was either extracted from dried young leaves of *Arctium lappa* L. (voucher HBH071) as previously reported (Idres et al. 2021) or chemically synthesized via stereoselective Michael 1,4‐addition (Matsuda et al. 2000; detailed protocol in Idres et al. 2021). PRE‐084 and PK11195 were purchased from Sigma‐Aldrich (France), dissolved in saline, and administered intraperitoneally (IP) at 100 μL/20 g body weight. Amyloid‐β[25–35] (Aβ_25‐35_) and its scrambled control peptide (Sc.Aβ) were obtained from Eurogentec, dissolved in sterile water (3 mg/mL), incubated 4 days at 37°C for oligomerization, and injected intracerebroventricularly (ICV) under isoflurane anesthesia as previously described (Maurice et al. 1996, 2019).

### 2.3 Experimental Design

**FIGURE 1:**
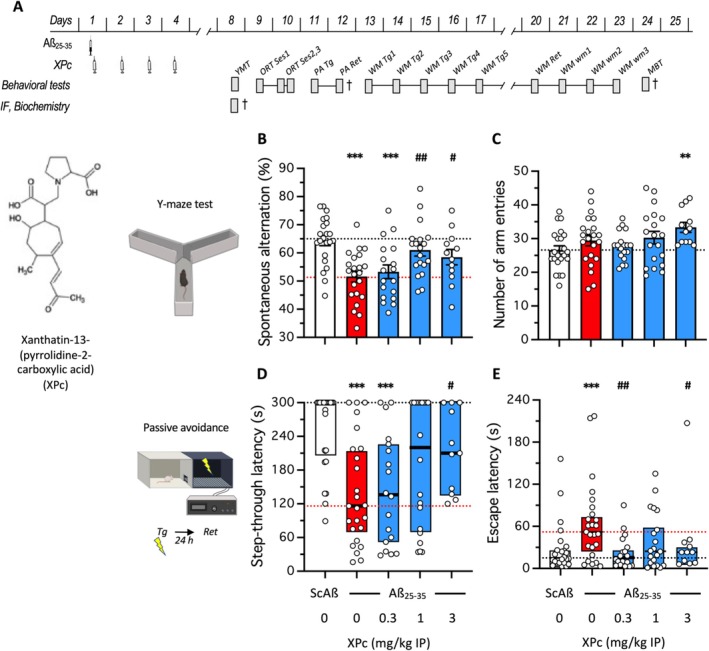
Neuroprotective effect of XPc on learning impairments induced by Aβ_25‐35_ ICV administration in mice: (A) Experimental protocol. Animals received Aβ_25‐35_ (9 nmol/3 μL, ICV) on day 1 and XPc (0.1–3 mg/kg IP) once‐a‐day between day 1 and 4. Animals were then analyzed using different behavioral tests between day 8 and day 13. YMT, Y‐maze test; MBT, marble burying test; PA, passive avoidance test; ORT, object recognition test; WM, water‐maze test; WM wm, water‐maze working memory test; Ses1~3, session 1~3; Tg, training session; Ret, retention session; †, euthanasia and brain dissection; IF, immunofluorescence analysis. (B) Spontaneous alternation and (C) total number of arm entries during the Y‐maze test; (D) step‐through latency and (E) escape latency in the passive avoidance test. Retention was measured 24 h after training for passive avoidance. Bar graphs show mean ± SEM and individual data in (B, C) and box‐and‐whisker graphs show median and interquartile range and individual data in (D, E). The numbers of animals per groups were: *N* = 12–22 in (B, C), *n* = 11–25 in (D, E). **p < 0.01, ***p < 0.001 versus (ScAß/V)‐treated group; # *p* < 0.05, ## *p* < 0.01 versus (Aß_25‐35_/V)‐treated group; Dunnett's test in (B, C), Dunn's test in (D, E).

Mice first received XPC (once daily for 4 consecutive days) before undergoing a battery of behavioral tests (Figure 1A). Three independent cohorts were used. Batch 1: mice were assessed for spatial working memory (Y‐maze spontaneous alternation), object recognition memory (novel object recognition test), and long‐term contextual memory (passive avoidance), followed by biochemical analyses. Batch 2: mice were assessed for spatial reference memory (water‐maze place learning) and anxiety‐related behavior (marble burying), followed by euthanasia. Batch 3: mice were assessed for spontaneous alternation only, followed by brain fixation for neuroinflammation immunofluorescence.

In combination studies, XPC, PK11195, and/or PRE‐084 were administered i.p. 20 min after Aβ_25‐35_. One week later, spontaneous alternation and passive avoidance were assessed over 3 days.

### 2.4 Behavioral Procedures

*Spontaneous alternation in the Y‐maze*. The test measures spatial working memory100 (Maurice et al. 1996; Meunier et al. 2006; Villard et al. 2011). In brief, mice explored a grey PVC Y‐maze (arms: 40 × 13 × 3–10 cm) for 8 min. Alternation percentage was calculated as (actual alternations / (total entries −2)) × 100. Mice with < 10 entries or alternation < 25% or > 90% were excluded (2.9% attrition).

*Novel object test*. The test assessed short‐term recognition memory, general behavior, and anxiety in session 1 (Maurice et al. 2019). In session 1, animals were allowed to acclimate during 10 min. After 24 h, mice explored two identical objects (session 2), then one familiar and one novel object 1 h later (session 3). The preference index was calculated as contacts (or time) with novel object/total contacts (or time). Animals with < 10 total contacts or no exploration of one object were excluded (2.8% attrition).

*Place learning in the water‐maze*. The test assessed spatial reference memory, with the fixed platform position learning protocol during days 1–8, and spatial working memory, with the daily changing platform location protocol during days 9–11 (Maurice et al. 2013). In brief, during fixed‐position platform training (days 1–8), mice did 3 trials/day, 90 s max, with 20 min ITI. The probe test was performed 72 h after the last training session. During the random‐position platform protocol (days 9–11), mice did 5 trials/day, new platform location each day, 2 min ITI. Mice never finding the platform unassisted were excluded (1.7% attrition).

*Marble burying test*. The test assessed anxiety levels. Fifteen (3 rows of 5) flat glass marbles were uniformly disposed in a cage filled with clean sawdust. Each mouse was placed in the center of the cage. After 30 min, the animal was removed and the level of marble burying was counted, with a score =2 for fully visible, =1 for half buried, and =0 for invisible marbles.

*Step‐through type passive avoidance*. The test assessed long‐term non‐spatial memory (Maurice et al. 2013, 2019). The apparatus was two black or white compartments (15 × 20 × 15 cm high) in a box. During training, mice entered the dark compartment and triggered a 0.3 mA, 3‐s foot‐shock. Retention was tested after 24 h and the latency to step into the dark compartment was measured with a cut‐off duration at 300 s. Animals showing no shock sensitivity and latencies < 10 s were excluded (2.9% attrition).

### 2.5 Brain Processing and Immunofluorescence

Mice received buprenorphine (0.1 mg/kg s.c.) 30 min before anesthesia (ketamine/xylazine). Brains were perfused with saline and Antigenfix, post‐fixed 48 h, cryoprotected in 20% sucrose, and coronally sectioned (25 μm). Sections were immunostained overnight at 4°C with anti‐IBA1 (1:250, ref. 019–19,741; Wako) and anti‐GFAP (1:400, ref. G3893; Sigma‐Aldrich), followed by secondary Cy3 Affinipure F(ab’)_2_ fragment donkey anti‐rabbit IgG (H + L) antibody (1:400; ref. 711–166‐1523; Jackson Immunoresearch) or secondary Alexa Fluor Plus 488 goat anti‐mouse IgG (H + L) antibody (1:1000; ref. A‐11001 Thermofisher scientific), antibody and 4ˊ,6‐diamidino‐2‐phenylindole (DAPI) counterstaining (1:50,000; ref. 62,247; Thermofisher scientific). Images were acquired on a Zeiss fluorescence microscope and analyzed with ImageJ. Cell counts (*n* = 5 animals/group, 4–5 sections/animal) were expressed as percentage of vehicle + Sc.Aβ group. Internal counting variability was 11.8% ± 0.9% for GFAP and 10.8% ± 1.0% for IBA1 in PoDG, 13.4% ± 1.0% for GFAP and 10.1% ± 1.0% for IBA1 in Mol, and 9.7% ± 0.8% for GFAP and 10.9% ± 1.1% for IBA1 in Rad.

### 2.6 Biochemical Assays

Hippocampal tumor necrosis factor‐α (TNFα) and interleukin‐6 (IL‐6) levels were quantified by ELISA (SEA133MU for TNFα and SEA079MU for IL‐6, Cloud‐Clone). Lipid peroxidation in the cortex was measured using the xylenol orange method as described (Maurice et al. 2019). Nitrotyrosine was detected by Western blot (anti‐nitrotyrosine 1A6, 1:1000; ref. 05–233; Sigma‐Aldrich) with Stain‐Free normalization. Quantification was done using ImageJ2 by selecting a fixed smir area along lanes.

### 2.7 Combination Studies

To allow pertinent combination analyses, drugs were injected with the same protocol, once, 20 min before Aβ_25‐35_ peptide. Animals were then analyzed using spontaneous alternation and passive avoidance to consider the drug protective effect on two different types of memories, namely spatial short‐term and non‐spatial long‐term memories. Combinations were then tested by mixing the maximal non‐active (MnA) and/or minimal active (mA) doses for XPc + PK11195 or XPc + PRE‐084, tested alone, and combination indexes (CI) were determined. Protective effects were expressed as percentage protection (PP), according to our previously published analyses (Maurice 2016; Martins et al. 1986; Martin et al. 2022; Freyssin et al. 2024). Isobolographic analysis was used to determine synergy (CI < 0.8), additivity (CI≈1), or antagonism (CI > 1).

Experimentally, CI must be lower than 0.8 to identify synergy. All calculations are presented in the Tables S1 and S2.

### 2.8 Statistical Analyses

Analyses were done using Prism v9.0 (GraphPad Software). Data were analyzed using one‐way or two‐way ANOVA, followed by a Dunnett's test. Passive avoidance latencies, expressed as median and interquartile range and represented as box‐and‐whiskers, were analyzed using a nonparametric Kruskal‐Wallis ANOVA and post hoc comparisons done using Dunn's test. Novel object preferences were analyzed versus 50% level (no recognition) using a one‐column *t*‐test. Significance level was *p* < 0.05. Full statistical details are provided in the Table S3.

## 3 Results

### 3.1 XPc Prevented Aβ_25‐35_ Peptide‐Induced Memory Impairments in Mice

Memory deficits and brain toxicity develop within 1 week after the ICV administration of oligomerized Aβ_25‐35_ peptide in mice. Aβ_25‐35_‐treated mice indeed showed a highly significant deficit in spontaneous alternation that was prevented by the 4‐days treatment with XPc at the doses of 1 and 3 mg/kg IP (Figure 1B). The drug treatment tended to increase the locomotor or exploratory behavior of the animals, as the number of arm entries increased significantly at the highest dose (Figure 1C). XPc also allowed a dose‐dependent prevention of Aβ_25‐35_‐induced deficits in passive avoidance. The step‐through latency decrease was dose‐dependently attenuated, with a significant reversion at 3 mg/kg IP (Figure 1D). The peptide treatment also increased the escape latency measured after mice entered the dark compartment (Figure 1E). We observed that the XPc treatment prevented this increase at all doses tested but more or less significantly (Figure 1E).

**FIGURE 2:**
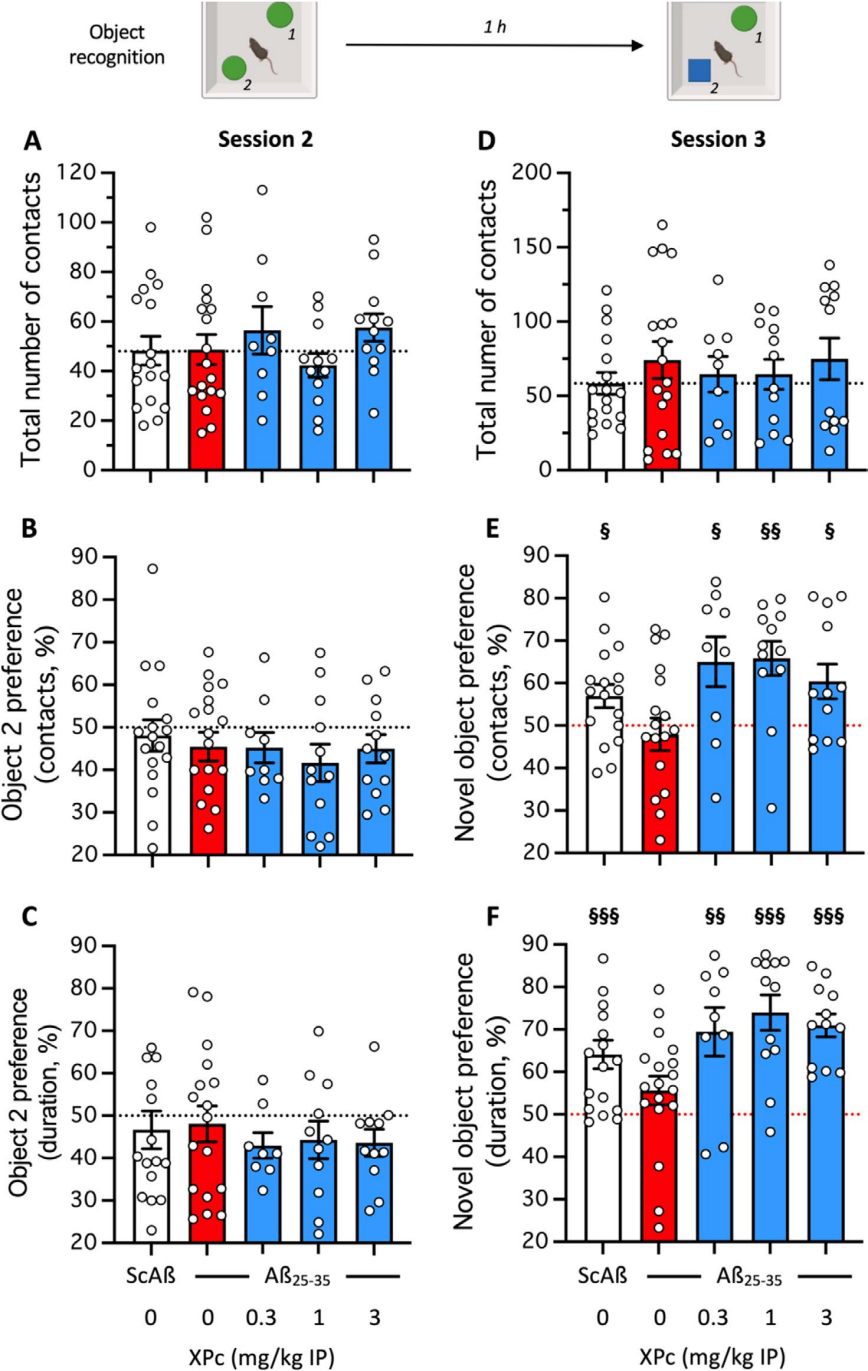
Neuroprotective effect of XPc on learning impairments induced by Aβ_25‐35_ ICV administration in mice: Object recognition test. (A–C) Session 2 with two identical objects, (D–F) session 3 with a novel and a familiar object; (A, D) Total number of contact with the objects, (B, E) preference for the object in position 2 or the novel object calculated from the number of contacts; (C, F) preference for the object in position 2 or the novel object calculated from the duration of contacts. Bar graphs show mean ± SEM and individual data. The numbers of animals per groups were: *N* = 12–18. ^§^ *p* < 0.05, ^§§^ *p* < 0.01, ^§§§^ *p* < 0.001 versus 50% value; one sample *t*‐test in (E, F).

In the novel object test, measuring recognition memory markedly altered in AD, the Aβ_25‐35_ or XPc treatment did not affect the global activity of the animals, measured during session 2 (with two similar objects) or session 3 (with a familiar and a novel object) using the total number of contacts with objects (Figure 2A,D). The global activity remained even similar between the two sessions with around 50 contacts whatever the group. During session 2, when the object exploration preference was analyzed in terms of number (Figure 2B) or duration (Figure 2C) of contacts with the objects, no difference between the two objects was noted whatever the treatment. During session 3, the V‐treated Aβ_25‐35_ group showed no preferential exploration of the novel object, contrarily to the V‐treated Sc.Aβ group, with preference calculated either in terms of number (Figure 2E) or duration (Figure 2F) of contacts. The XPc treatments, whatever the dose, restored a preferential exploration of the novel object for both parameters (Figure 2E,F).

**FIGURE 3:**
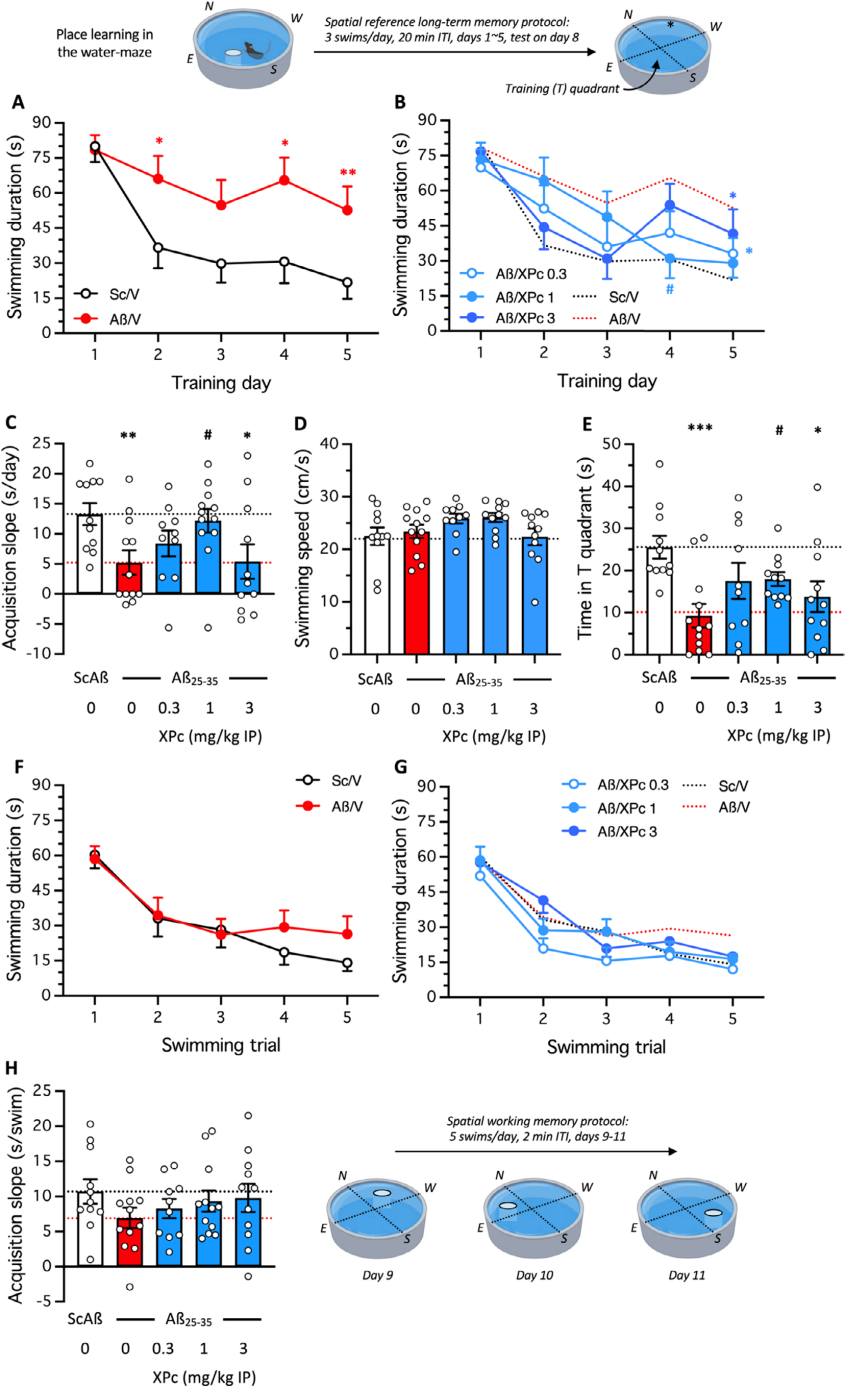
Neuroprotective effect of XPc on learning impairments induced by Aβ_25‐35_ ICV administration in mice: Place learning in the water‐maze. (A–E) Spatial reference memory protocol: Animals were trained by performing 3 swims per day with 20 min ITI during 5 days and memory retention of the platform location was checked after 72 h in a probe test. (A, B) Acquisition profiles and (C) acquisition slope during training; (D) swimming speed and (E) presence in training (T) quadrant during the probe test. (F‐H) Spatial working memory protocol: Platform location was changed every day and animals were trained by performing 5 swims per day with 2 min ITI during 3 days. (F, G) Acquisition profiles and (H) acquisition slope. The numbers of animals per groups were: *N* = 10–12. *p < 0.05, **p < 0.01, ***p < 0.001 versus ScAß/V‐treated group; ^#^ *p* < 0.05 versus Aß_25‐35_/V‐treated group; unpaired *t*‐test.

Spatial reference memory was analyzed using place learning in the water‐maze (Figure 3A–E). Mice had to find a fixed platform location during 5 days. V‐treated Sc.Aβ mice progressively learned the platform location, reaching a median latency about 20 s after 5 training days (Figure 3A). V‐treated Aβ_25‐35_ mice showed an impaired acquisition profile, with significant increases in swim duration on trials 2, 4, and 5 (Figure 3A). The XPc treatment improved the acquisition profiles as compared to the Aβ_25‐35_‐treated group. In particular, during trials 4–5, the 0.3 and 1 mg/kg doses, but not 3 mg/kg, led to decreased swim durations close to the control profile of V‐treated Sc.Aβ mice (Figure 3B). To further analyze the drug effect, acquisition slopes were determined. The treatment significantly decreased acquisition slopes from 14 s/day for the V‐treated Sc.Aβ group to 5 s/day for the V‐treated Aβ_25‐35_ group (Figure 3C). The XPc treatment attenuated in a bell‐shaped manner the slope diminution, with a significant effect at 1 mg/kg (Figure 3C). A probe test was performed 3 days after the last training trial, without platform in the pool and by measuring in a 1‐min swim, the time spent by each mouse in the training quadrant of the pool. Swim speed was evaluated during this probe test and was unaffected by the treatments (Figure 3D). As compared to the V‐treated Sc.Aβ group performance, the V‐treated Aβ_25‐35_ group showed a highly significant decrease of time spent in the T quadrant. This diminution was attenuated by the XPc treatment, again in a bell‐shaped manner and with a significant effect at 1 mg/kg (Figure 3E).

After the reference memory procedure, spatial working memory was tested in the water‐maze over 3 days, by changing daily the platform location and testing animals with a very tight (2‐min) ITI (Figure 3F–H). Surprisingly, the Aβ_25‐35_‐induced deficit in acquisition was moderate (Figure 3F), and although the XPc treated groups showed profiles closer to the V‐treated Sc.Aβ group (Figure 3G), no significant difference was measured. The analysis of acquisition slopes measured for this session confirmed a trend to a decrease (from 11 to 7 s) for the V‐treated Aβ_25‐35_ group and a trend to a dose‐dependent attenuation of this decrease by XPc (Figure 3H), data were not as clear‐cut as observed in the Y‐maze test, measuring a similar memory process.

**FIGURE 4:**
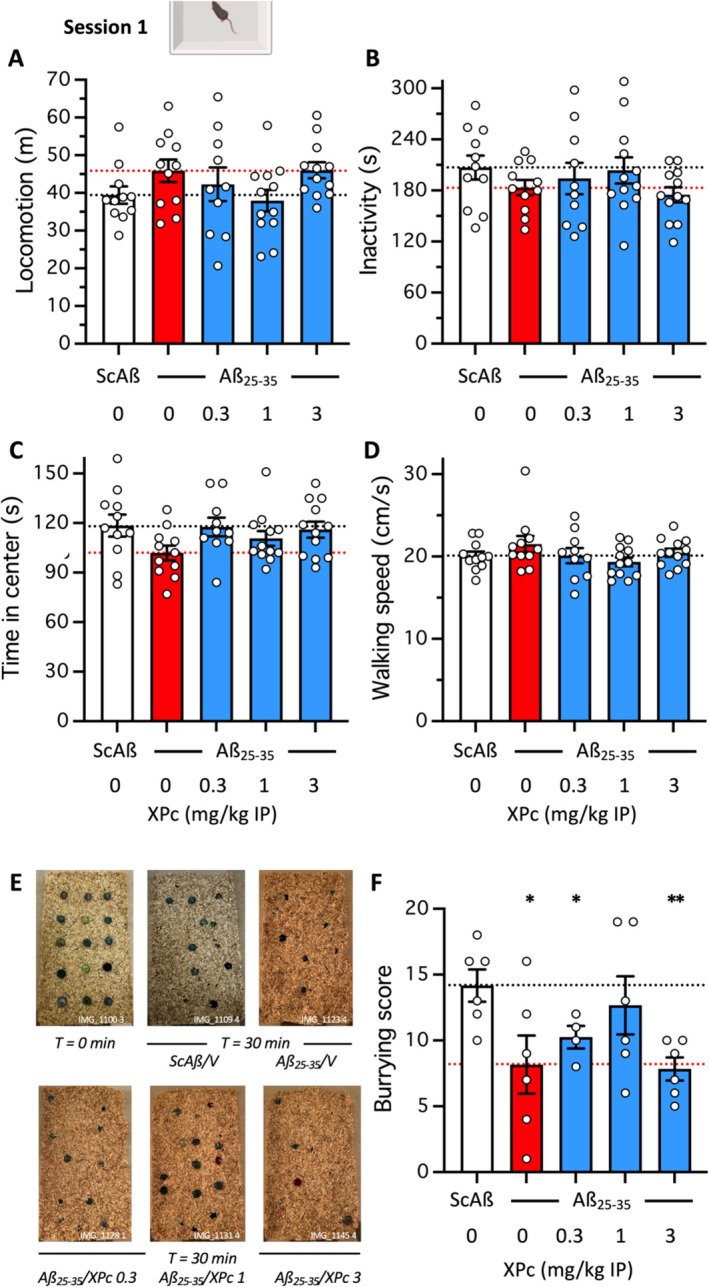
Effect of XPc in the (A‐D) session 1 (open‐field) of the novel object test and (E, F) marble burying test in Aβ_25‐35_‐treated mice. (A) total locomotion, (B) inactivity duration, (C) time in center, and (D) walking speed during the 10 min session in the square open‐field during the session 1 of the object recognition test. (E) Typical pictures of the marbles disposed in the cage before (*t* = 0 min) and after (T = 30 min) the burying session for individual of each treatment group. (F) Burying scores. *p < 0.05, **p < 0.01 versus (ScAß/V)‐treated group; Dunnett's test in (F).

The behavioral analyses assessed different memory processes in Aβ_25‐35_‐treated mice and showed that XPc attenuated the deficits in the 0.3–3 mg/kg dose‐range with mostly a bell‐shaped effect culminating at 1 mg/kg, therefore appearing to be the most active dose. Interestingly, the first session of the novel object test (without object in the arena) allowed an open‐field analysis of exploratory activity and anxiety. We completed these analyses with a marble burying test, directly assessing the anxious status of the animals. The data from session 1 of the novel object test revealed only trends among groups. Locomotion during the 10‐min session tended to be increased by Aβ_25‐35_ (Figure 4A), while inactivity (Figure 4B) and the time in the center of the arena (Figure 4C) tended to be decreased, suggesting moderate hyperactivity and anxiety. The XPc treatment tended to prevent these effects. Interestingly, the walking speed, related to exploration but also dependent on motor problems, was unaffected by the treatments (Figure 4D). The marble burying test, however, showed a significant default in burying the marbles for the V‐treated Aβ_25‐35_ group, that was attenuated in a bell‐shaped manner by the XPc treatment and with 1 mg/kg being the most active dose (Figure 4E,F). The marble burying test data therefore suggested that XPc allowed not only an anti‐amnesic effect but also anti‐anxiety, so, more generally, a cognitive improvement in Aβ_25‐35_‐treated mice.

### 3.2 The XPc Treatment Prevented Neuroinflammation and Oxidative Stress in the Brain of Aβ_25‐35_‐Treated Mice

**FIGURE 5:**
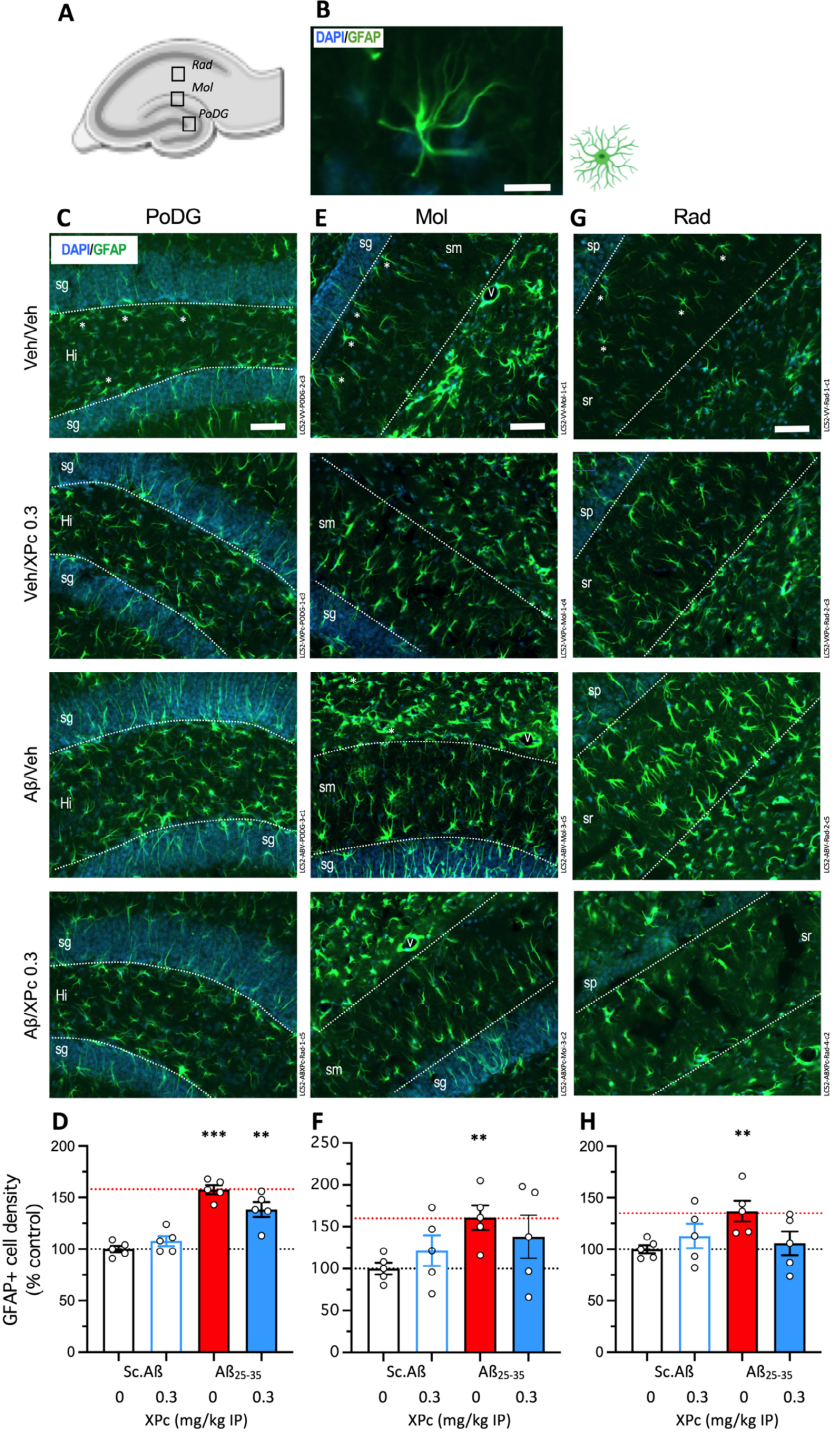
Protective effects of XPc on astroglial reaction in the hippocampus of Aβ_25‐35_‐treated mice. (A) Delimitation of the three glial areas analyzed in hippocampal formation: *Stratum radiatum* (Rad); *stratum moleculare* (Mol) and polymorph layer of the dentate gyrus (hilus). (B) Astroglial cell were immunolabeled with GFAP and cell bodies with DAPI: Higher magnification of a typical GFAP‐labeled astrocyte. (C, E, G) Typical immunofluorescence micrographs of coronal 25 μm thick sections (blue: DAPI, green: GFAP) and (D, F, H) quantifications of the density of GFAP+ immunofluorescent cells, expressed as percentage of the V‐treated Sc.Aβ group data. (C, D) Polymorph layer of the dentate gyrus (PODG); (E, F) *stratum moleculare* (MOL); and (G, H) *stratum radiatum* (RAD). 4–5 slices were counted per animal and the value averaged for each area. Sp., *stratum pyramidale*; sr, *stratum radiatum*; sg, *stratum granulare*; sm, *stratum moleculare*; Hi, *hilus*; V, capillary vessel. Scale bars in (C, E, G) = 50 μm. *p < 0.05, **p < 0.01, ***p < 0.001 versus V‐treated Sc.Aβ group, Dunnett's test.

**FIGURE 6:**
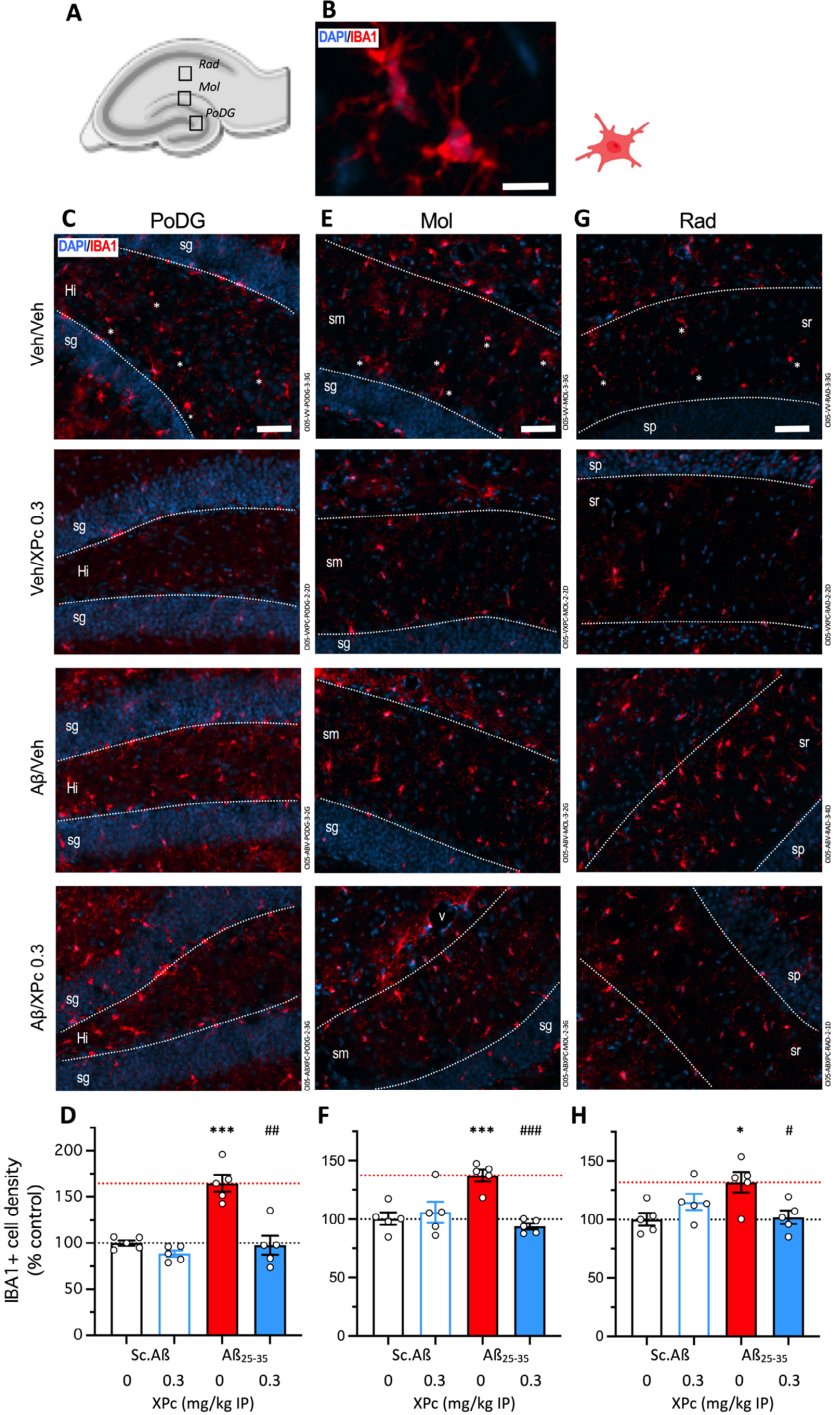
Protective effects of XPc on microglial reaction in the hippocampus of Aβ_25‐35_‐treated mice. (A) Delimitation of the three glial areas analyzed in hippocampal formation: *Stratum radiatum* (Rad); *stratum moleculare* (Mol) and polymorph layer of the dentate gyrus (hilus). (B) Microglial cell were immunolabeled with IBA1 and cell bodies with DAPI: Higher magnification of typical IBA1‐labeled microglia. (C, E, G) Typical immunofluorescence micrographs of coronal 25 μm thick sections (blue: DAPI, red: IBA1) and (D, F, H) quantifications of the density of IBA1+ immunofluorescent cells, expressed as percentage of the V‐treated Sc.Aβ group data. (C, D) Polymorph layer of the dentate gyrus (PODG); (E, F) *stratum moleculare* (MOL); and (G, H) *stratum radiatum* (RAD). 4–5 slices were counted per animal and the value averaged for each area. Sp., *stratum pyramidale*; sr, *stratum radiatum*; sg, *stratum granulare*; sm, *stratum moleculare*; Hi, *hilus*; V, capillary vessel. Scale bars in (C, E, G) = 50 μm. *p < 0.05, ***p < 0.001 versus V‐treated Sc.Aβ group; ^#^ *p* < 0.05, ^##^ *p* < 0.01, ^###^ *p* < 0.001 versus V‐treated Aβ_25‐35_ group, Dunnett's test.

The brain of mice was then examined for toxicity, neuroinflammation and oxidative stress. Neuroinflammation was first analyzed using astroglial (Figure 5) and microglial (Figure 6) reactions. Three glial areas essential for the hippocampal neuronal connectivity were analyzed: the polymorph layer, or hilus, in the dentate gyrus (PoDG), the molecular layer (Mol) and radial layer (Rad) (Figure 5A). Reactive astroglia was analyzed using immunofluorescent labeling (IF) of GFAP (Figure 5B). As compared to V‐treated Sc.Aβ mice, the Aβ_25‐35_ treatment increased the density of GFAP‐positive cells significantly in the PoDG (+50%, Figure 5C,D) and Mol (+60%, Figure 5E,F) but only moderately in the Rad (+35%, Figure 5G,H). The XPc treatment, at 1 mg/kg IP, failed to affect GFAP IF in all three areas, although a decreasing trend was noted in PoDG (*p* = 0.052 vs. V‐treated Aβ_25‐35_ group; Figure 5D) and in Rad (*p* = 0.076 vs. V‐treated Aβ_25‐35_ group; Figure 5H).

In the same 3 glial areas (Figure 6A). Reactive microglia were analyzed using IF of IBA1 (Figure 6B). As compared to V‐treated Sc.Aβ mice, the Aβ_25‐35_ treatment significantly increased the density of IBA1‐positive cells in the PoDG (+65%, Figure 6C,D) and Mol (+37%, Figure 6E,F), and in the Rad (+32%, Figure 6G,H). The XPc treatment, at 1 mg/kg IP, significantly blocked IBA1 IF in all three areas (Figure 6C–H), showing a very efficient protection on Aβ_25‐35_‐induced microglial activation.

**FIGURE 7:**
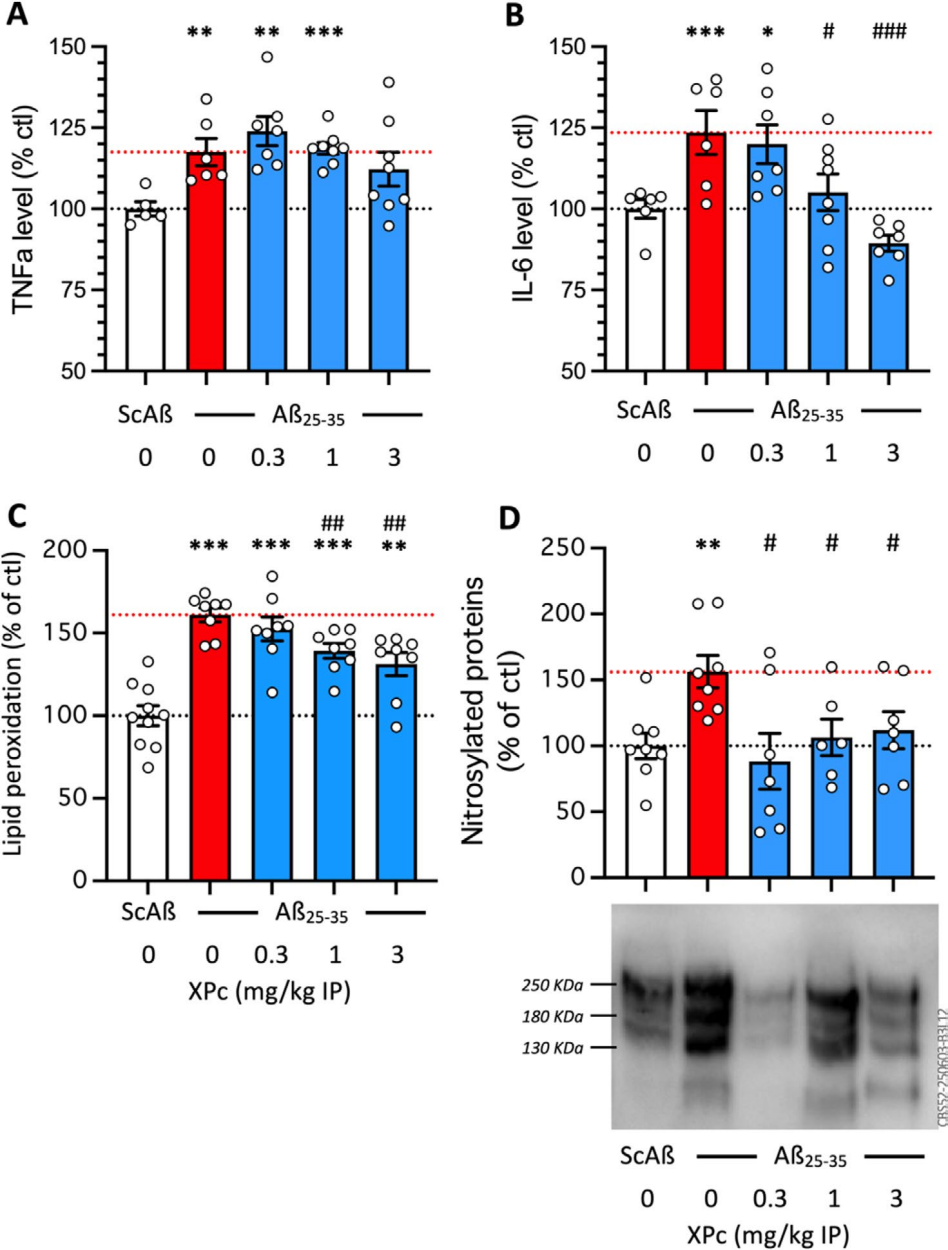
Protective effet of XPc on Aβ_25‐35_‐induced neuroinflammation and oxidative stress: Levels in (A) TNFα, (B) interleukin‐6 (IL‐6), (C) lipid peroxidation, and (D) nitrosylated proteins, in the mouse hippocampus. Animals received Aβ_25‐35_ (9 nmol/mouse ICV) on day 1, XPc (0.1–1 mg/kg IP) once‐a‐day between days 1 and 4, and animals were sacrificed on day 8. TNFα and IL‐6 contents were analyzed by ELISA, lipid peroxidation using the cumene peroxide colorimetric assay, and nitrosylated proteins by a nitro‐Tyr western blot assay. Data show mean ± SEM. Experiments were repeated 3 times. A typical blot is shown in D. *p < 0.05, **p < 0.01, ***p < 0.001 versus V‐treated Sc.Aβ group; # *p* < 0.05, ## *p* < 0.01, ### *p* < 0.001 versus V‐treated Aβ_25‐35_ group; Dunnett's test.

We also analyzed the global content in two cytokines in hippocampal tissue preparation by Elisa: TNFα, that is released by and priming astrocytes (Abd‐El‐Basset et al. 2021; Heir et al. 2024), and IL‐6, playing the same role on microglia (Garner et al. 2018; Sanchis et al. 2020). The Aβ_25‐35_ treatment significantly increased TNFα in hippocampal tissue (+18%; Figure 7A), but the XPc treatment only marginally affected this increase, only non‐significantly at the highest dose tested. The Aβ_25‐35_ treatment significantly increased IL‐6 level in the tissue (+24%; Figure 7B), and the XPc treatment dose‐dependently attenuated this increase with significant differences at 1 and 3 mg/kg. These tissue analyses therefore appeared coherent with the IF analyses of glial reaction.

Oxidative stress is induced after Aβ_25‐35_ treatment. We analyzed two parameters measuring consequences of oxidative stress on lipids and proteins in hippocampal preparations: the level of lipid peroxidation of the cell membranes (Figure 7C), using a sensitive colorimetric assay, and the level of protein nitrosylation (Figure 7D), using a Western blot approach. The Aβ_25‐35_ treatment significantly increased lipid peroxidation (+61%; Figure 7C) and the XPc treatment dose‐dependently but partially attenuated the increase with significant differences at the doses of 1 and 3 mg/kg. The Aβ_25‐35_ treatment significantly increased protein nitrosylation (+52%; Figure 7D), an effect that was blocked by the XPc treatment at each dose tested. These measures confirmed the potent anti‐oxidant effect of XPc, which primarily acts as a G6PDH activator and therefore as an antioxidant targeting glutathione peroxidase (Idres et al. 2021).

### 3.3 XPc Showed Synergistic Effects With the TSPO Activator PK11195 or the Sigma‐1 Receptor Agonist PRE‐804

**FIGURE 8:**
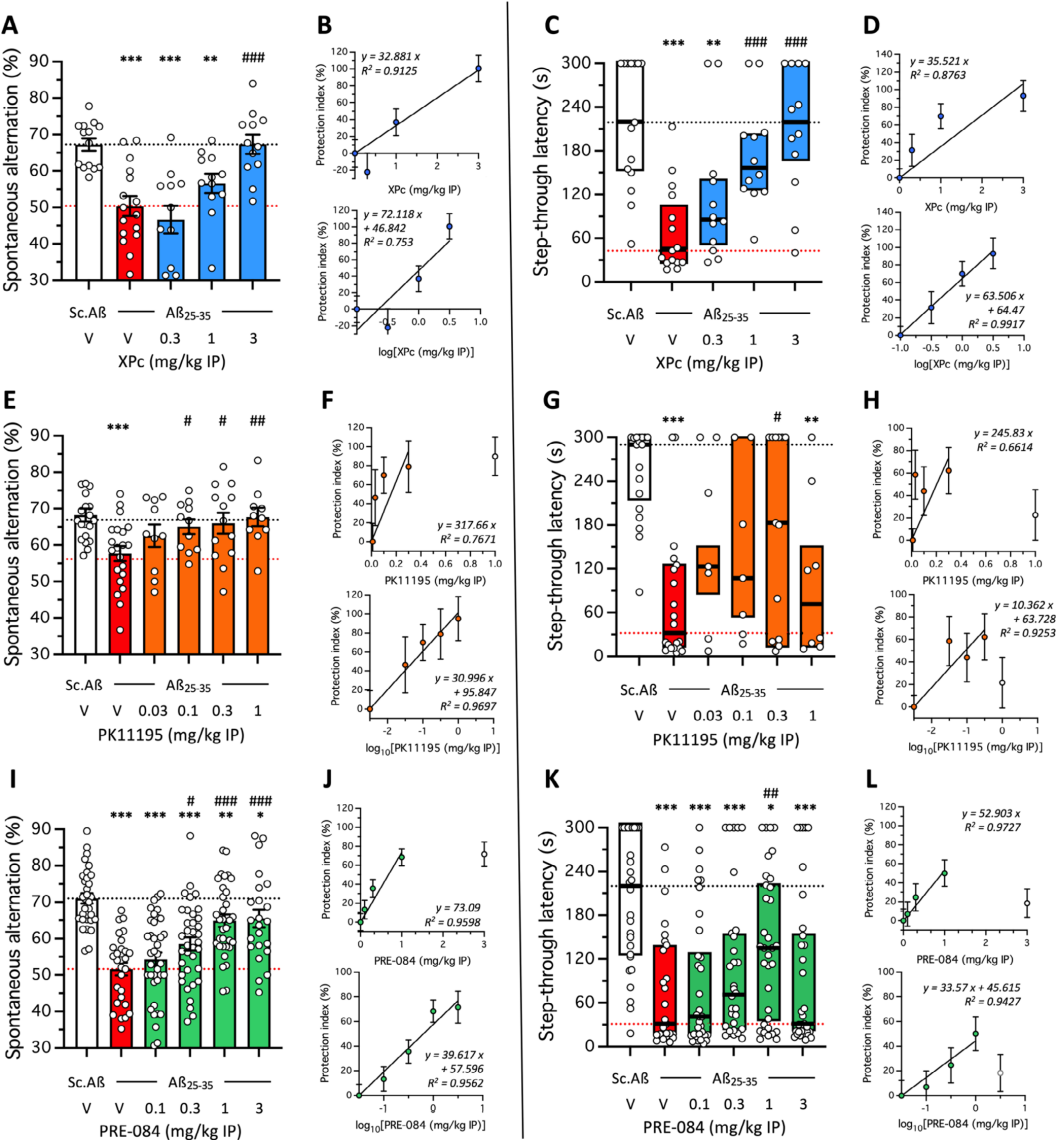
Dose–response effect of (A‐D) XPc (0.3–3 mg/kg IP), (E‐H) PK11195 (0.03–1 mg/kg IP), and (I‐L) PRE‐084 (0.1–3 mg/kg IP) against Aβ_25‐35_‐induced learning impairments in mice. Animals received the drugs on day 1 20 min before Aβ_25‐35_ (9 nmol ICV). The Y‐maze test session was performed on day 8 and passive avoidance training was performed on day 9. Retention was analyzed on day 10, 24 h after training. Left panel: Spontaneous alternation in the Y‐maze. Right panel: Passive avoidance latencies. Data show mean ± SEM in (A, E, I) and median and interquartile range in (C, G, K). In (B, D, F, H, J, L), the protection index was calculated with reference values based on the V‐treated Sc.Aβ group data as 100% and V‐treated Aβ_25‐35_ group data as 0% and regression analyses presented as linear or log_10_. *p < 0.05, **p < 0.01, ***p < 0.001 versus V‐treated Sc.Aβ group; ^#^ *p* < 0.05, ^##^ *p* < 0.01, ^###^ *p* < 0.001 versus V‐treated Aβ_25‐35_ group; Dunnett's test in (A, E, I), Dunn's test in (C, G, K).

**FIGURE 9:**
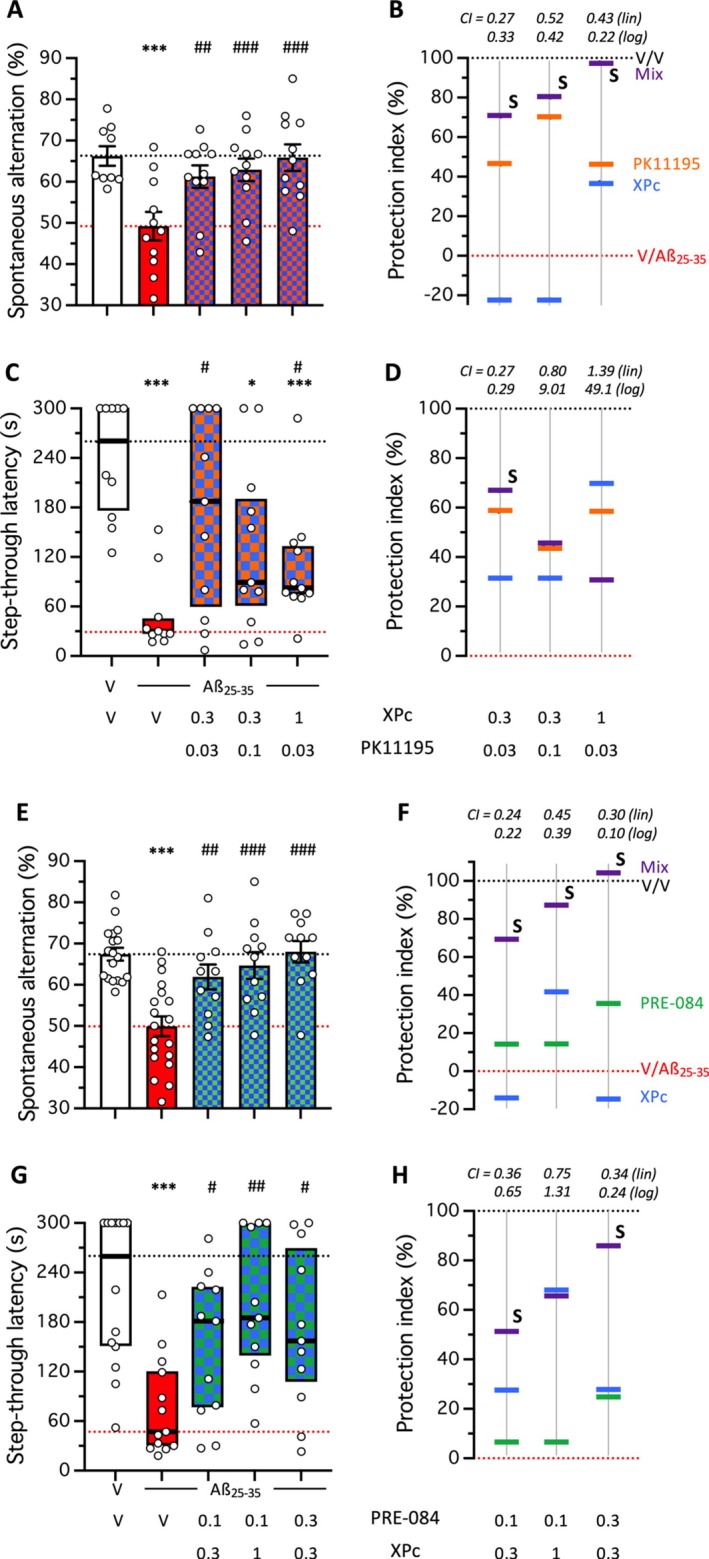
Combination studies between (A‐D) XPc and PK11195, and (E‐H) XPc and PRE‐084 in Aβ_25‐35_‐treated mice. (A, B, E, F) Spontaneous alternation in the Y‐maze; (C, D, G, H) step‐though passive avoidance. Animals received the drugs on day 1 20 min before Aβ_25‐35_ (9 nmol ICV). The Y‐maze test session was performed on day 8 and passive avoidance training was performed on day 9. Retention was analyzed on day 10, 24 h after training. Data show mean ± SEM in (A, E) and median and interquartile range in (C, G). *p < 0.05, ***p < 0.001 versus V‐treated Sc.Aβ group; ^#^ *p* < 0.05, ^##^ *p* < 0.01, ^###^ *p* < 0.001 versus V‐treated Aβ_25‐35_ group; Dunnett's test in (A, E); Dunn's test in (C, G). In (B, D, F, H), protection is shown using a cursor‐on‐scale representation, with reference values based on the V‐treated Sc.Aβ group data as 100% and V‐treated Aβ_25‐35_ group data as 0%. Calculated protection indexes are detailed in Table S1 (spontaneous alternation) and Table S2 (passive avoidance). Mix, XPc + PK11195 or XPc + PRE‐084; CI, combination index, calculated from the linear or log_10_ regression analysis; S, synergistic effect with combination index (CI) < 1.

The protocol for combination studies in the Aβ_25‐35_‐induced AD model has been previously described by us (Maurice 2016; Freyssin et al. 2024) and requires first a dose–response study of each drug alone to determine the regression equations for each drug (Figure 8; Tables S1 and S2), and then analysis of the combinations based on mix of the MnA and/or mA dose of each drug (Figure 9; Tables S1 and S2). The combination also requires a similar administration protocol for each drug, although the precise pharmacokinetics of each drug are not yet known. We administered the drugs once, 20 min after the Aβ_25‐35_ peptide on day 1 and performed the Y‐maze test on day 8 and passive avoidance on days 9 (training) and 10 (retention). XPc showed a dose‐dependent protection against Aβ_25‐35_‐induced learning deficits under this protocol with the active doses of 3 mg/kg in the Y‐maze (Figure 8A) and 1 and 3 mg/kg in passive avoidance (Figure 8C). Both linear and logarithmic regressions were calculated in the two behavioral responses and showed coefficients *R* ^2^ between 0.75 and 0.99 (Figure 8B,D; Tables S1 and S2). This indicated that the accuracy to the behavioral data slightly varied according to the mode of calculation and justified the dual calculation and analysis. PK11195 also attenuated Aβ_25‐35_‐induced memory deficits in the 0.1–1 mg/kg dose range for spontaneous alternation (Figure 8E) and at 0.1 and 0.3 mg/kg for passive avoidance (Figure 8G). Regression calculations led to R^2^ between 0.66 and 0.97 (Figure 8F,H; Tables S1 and S2). Finally, PRE‐084 also attenuated Aβ_25‐35_‐induced memory deficits in the 0.3–3 mg/kg dose range for spontaneous alternation (Figure 8I) and at 1 mg/kg for passive avoidance (Figure 8K). *R* ^2^ between 0.94 and 0.97 were determined for the regression calculations (Figure 8B,D; Tables S1 and S2). Note that the highest dose could be excluded from the calculation (for PK11195 and PRE‐084) when a bell‐shaped dose–response curve was observed.

These data also allowed to define the MnA and mA doses and test the combinations (Figure 9). The XPc + PK11195 combinations led to significant protection in the Y‐maze (Figure 9A) and passive avoidance (Figure 9C) at almost all doses tested. The protection index representations (Figure 9B,D) and calculation of combination index (Tables S1 and S2) showed that all combinations tested led to synergistic protection in the short‐term memory test (Figure 9B) and for the low dose combination in the long‐term memory test (Figure 9D). The XPc + PRE‐084 combinations led to significant protection at all doses tested in the Y‐maze (Figure 9E) and passive avoidance (Figure 9G) at almost all doses tested. The protection index representations (Figure 9F,H) and calculation of combination index (Tables S1 and S2) showed that all combinations tested led to synergistic protection in the short‐term memory test (Figure 9F) and for the low and high dose combinations in the long‐term memory test (Figure 9H). These observations demonstrated the synergistic potencies of drug combinations based on XPc with other anti‐inflammatory and anti‐oxidant drugs.

## 4 Discussion

We report that XPc, a sesquiterpene lactone isolated from Burdock leaf (*Arctium lappa L*.) (Idres et al. 2021), is protective at low doses in a pharmacological mouse model of AD. The sesquiterpenoid effects were previously shown on biochemical markers of the oxidative stress and neuroinflammation (Matos et al. 2021; La et al. 2024; Yang et al. 2025; Cutter et al. 2025). Among them, the sesquiterpene lactones are widely studied for their ability to target and inhibit pro‐inflammatory factors responsive the inflammatory process (Mazor et al. 2000; Matos et al. 2021; Ivanescu et al. 2015). Among various sesquiterpene lactones isolated from Artemisia (*Artemisia argyi*), six of them exhibited a strong anti‐neuroinflammatory effect, particularly argyinolide S (La et al. 2024). This compound exerted anti‐inflammatory effect via the inhibition of the JAK1/STAT3 pathway (Li et al. 2023) in mouse microglial BV‐2 cells. Moreover, different sesquiterpene lactones were shown to prevent behavioral modifications, amyloid toxicity in both in vitro and in vivo, and cognitive impairments in AD models (Amoah et al. 2015; Tang et al. 2022). For instance, alantolactone and isoalantolactone prevented the cytotoxicity of Aβ_25‐35_ (10 μM) in neuronal cells derived from the mouse cerebral cortex and inhibited the production of intracellular reactive oxygen species, including superoxide anion induced by Aβ_25‐35_ (Seo et al. 2017). Achilollide A, isolated from *Achillea fragrantissima*, decreased Aβ_25‐35_‐induced death of Neuro2a neuroblastoma cells (Elmann et al. 2018). In line with these observations, we investigated the XPc neuroprotective action through its preventive effect on Aβ_25‐35_‐induced memory deficits. In our work, we have used a battery of behavioral tests assessing spatial working memory, recognition memory, spatial reference memory, and non‐spatial contextual memory. The drug allowed a complete protection of the impairments measured in the different tests, therefore suggesting that it efficiently helped to protect synaptic connections and activity and cellular integrity in the brain structures involved in memory encoding and subjected to the Aβ_25‐35_ toxicity. The drug also attenuated Aβ_25‐35_‐induced anxiety in the marble burying test, thus more generally protected against the Aβ_25‐35_‐induced cognitive impairment. The active dose range was as low as 1 mg/kg IP, indicating that XPc appeared as active as the reference neuroprotective drugs like donepezil or other cholinesterase inhibitors (Meunier et al. 2006), memantine (Couly et al. 2021) or the investigational drug blarcamesine (Villard et al. 2011), that were tested using the same administration route and pharmacological model in mice. This indicated that the sesquiterpene lactone XPc has a very good bioavailability in mice and, although it has still to be precisely examined in other species and humans, a promising translational profile for further clinical development. In previous studies, alantolactone and isoalantolactone used in in vivo experiments reversed the cognitive impairments caused by scopolamine as assessed in the water‐maze, Y‐maze and passive avoidance tests. The compounds decreased acetylcholinesterase activities in a dose‐dependent manner (Seo et al. 2017). Under chronic treatment in transgenic mouse models, 15‐acetoxy‐isogermafurenolide, 15‐hydroxy‐isogermafurenolide, podoandin, 1,2‐epoxy‐10α‐hydroxy‐podoandin, 13‐hydroxy‐8,9‐dehydroshizukanolide, and aromadendrane‐4β,10α‐diol significantly ameliorated the Aβ_1‐42_‐induced passive avoidance impairments, when co‐administered ICV with Aβ_1‐42_ in mice (Amoah et al. 2015). Dihydroartemisinin, the active metabolite of artemisinin, improved the cognitive impairment of APP/PS1 mice tested in the Barnes maze, after a 3‐month treatment *per os* by gavage (PO) (Zhao et al. 2020). A 21‐days treatment with 1,6‐*O,O*‐diacetylbritannilactone attenuated the impairments in cognitive function observed in 6‐month‐old 5xFAD mice, assessed using the Morris water‐maze test (Tang et al. 2022). We therefore confirmed the potentialities of this class of natural compounds in neurodegenerative diseases. By using an acute model, we could show that only a 4‐days treatment, or even a single injection in the combination studies, was able to induce significant protection against Aβ_25‐35_ toxicity. It must be noted that both dihydroartemisinin and 1,6‐*O,O*‐diacetylbritannilactone were used at a higher dose, 20 mg/kg PO or IP. Moreover, alantolactone was also tested in vivo in a *Porphyromonas gingivalis* ‐infection model in rats and significantly attenuated the memory deficits but also at a high dose range: 25–100 mg/kg PO for 6 weeks (Chen et al. 2024). A direct comparison between XPc and these lactones must be conducted to confirm its putative better in vivo potency among sesquiterpene lactones.

Among the toxicity markers we analyzed in the brain of Aβ_25‐35_‐treated mice, we measured the astroglial and microglial reactions by immunofluorescence in hippocampal slices. Interestingly, we observed that XPc attenuated astroglial reaction but completely prevented microglial reaction, with significant prevention measured in all 3 areas analyzed of the hippocampus. Global tissue analysis of cytokines levels using Elisa also showed that the increase in IL‐6, mainly released by microglia, was blocked, while the increase in TNFα, mainly released by astroglia, was only attenuated at the highest dose tested. These observations indicated a preferential anti‐inflammatory effect of XPc targeting microglia. Such differential effect has been previously reported particularly in in vitro models (Galvin et al. 2025), but not in in vivo models where the long‐duration chronic treatment finally permitted a complete protection for both astroglial and microglial reactions (Tang et al. 2022; Chen et al. 2024). The protective effect of sesquiterpene lactones has indeed been largely studied in vitro, using the BV‐2 microglial cell line activated using lipopolysaccharide (LPS) or lipoteichoic acid (LTA) (Wang et al. 2018; Tang et al. 2022; Galvin et al. 2025). BV‐2 microglial cells released massively IL‐6 (30× increase in cell medium) and much lower TNFα (5–6× increase) under LTA or LPS challenge. Ergolide was shown to more significantly decrease IL‐6 levels than TNFα levels (Galvin et al. 2025). Other markers of microglial activation, including IL‐10 or NF‐κB, were attenuated by sesquiterpene lactones and the protective pathways identified in several studies using this cell line.

Besides their anti‐inflammatory effects, some sesquiterpene lactones are known for their antioxidant properties (Shoaib et al. 2017; Wang et al. 2022; El Khatib et al. 2021). Some different sesquiterpene lactones from *Calea urticifolia*, parthenolide from *Tanacetum parthenium* (Pooja and Shetty 2021), vernomelitensin and onopordopicrin from *Onopordum illyricum* (Umemura et al. 2008) and hemistepsin from *Hemistepta lyrata* (Kim et al. 2018) have been described as nuclear factor‐erythroid‐2‐related factor 2 (Nrf2) activators and are able to elicit increased resistance to oxidative stress (Formisano et al. 2017). The Nrf2 activation is done through the dissociation from Kelch‐like ECH‐associated protein 1 (Keap1) (Alonso et al. 2018). Being released, Nrf2 can enter the nucleus to engage in transcription of detoxification enzymes such as heme oxygenase‐1 (HO‐1), SOD, GPx, and GST, and thus contribute to the antioxidant defense mechanism (Umemura et al. 2008). In the previous work, the L6 muscular cells‐XPc induced did not show any modifications on the transcriptional activity of the SOD and GPx genes. The hypothesis is that XPc stimulates G6PD enzymatic activity through a direct molecular interaction at the site previously mentioned to stabilize the G6PD dimer in its enzymatically active configuration (Idres et al. 2021). An increase in G6PD activity leads to glutathione (GSH) regeneration by reduction of oxidized glutathione (GSSG) and also produces nicotinamide adenine dinucleotide phosphate (NADPH), a high electron donor for reductive biosynthesis and antioxidant defense (Salvemini et al. 1999). NADPH acts as a cofactor of NADPH‐oxidase enzymes (NOx), nitric oxide synthase (NOS), dihydrofolate reductase (DHFR), and cytochrome P450 oxidoreductase. Therefore, activating the G6PD enzyme is particularly important in the context of AD as it reinforces the antioxidant defense. Increased cerebral G6PD enzyme activity, targeted by XPc, is observed in AD (Martins et al. 1986) and directly related to the extent of oxidative stress. Moreover, G6PD overexpression in *Drosophila melanogaster* and mice acts as protection against oxidative damage and age‐associated functional decline (Nóbrega‐Pereira et al. 2016). To accurately evaluate the G6PD activator effect of XPc, it is necessary to measure precisely the various GSH/GSSG and NADPH/NADP+ ratios. Furthermore, it is necessary to study the impact of XPc on the enzymes involved in the GSH synthesis as well as all enzymes NADPH‐dependent involved in oxidative stress response. To accomplish this goal, we will carry out a transcriptomic study.

In AD context, cellular antioxidant defenses are altered, oxidative damage at the cellular level is increased, neuronal levels and cognitive functions are impaired as observed in AD mouse models. So, the Nrf2 pathway which promotes the transcription of a large number of genes that are implicated in oxidative stress defense and redox imbalance is also important. Activation of the Nrf2 pathway is also likely involved in the synergic effect observed in combination studies. Indeed, we first combined XPc with a sigma‐1 receptor agonist, PRE‐084, that has been shown to exert potent anti‐oxidant, anti‐inflammatory and anti‐apoptotic effects in several preclinical models of neurodegenerative diseases (Maurice et al. 1999; Francardo et al. 2014; Motawe et al. 2020; Crouzier et al. 2022; Lasbleiz et al. 2022), including Aβ_25‐35_‐induced toxicity in mice (Maurice et al. 1994). S1R agonists exert their cytoprotective effect by restoring numerous intracellular pathways related to calcium homeostasis among organelles (Hayashi and Su 2007) or stabilizing numerous receptor‐mediated signaling (Su et al. 2016). In particular, the S1R agonist cutamesine (SA4503) treatment attenuated lipopolysaccharide (LPS)‐induced inflammatory reactions and oxidative/nitrosative stress by downregulating the expression of iNOS and TNF‐α, and upregulating GSH in cultured astrocytes by increasing Nrf2 and HO‐1 expression (Wang and Zhao 2019). In a zebrafish model of amyotrophic lateral sclerosis overexpressing a mutant TDP43 protein, we reported that PRE‐084 increased binding immunoglobulin protein (BiP) levels and eukaryotic initiation factor 2α (EIF2α)/activating transcription factor 4 (ATF4) and Nrf2 signaling cascades, suggesting that S1R PRE‐084 prevents mutant TAR DNA‐binding protein 43 (TDP43) toxicity by boosting ER stress response and antioxidant cascade through Nrf2 signaling (Lasbleiz et al. 2022). A similar observation was recently provided using the S1R agonist afobazole alleviating streptozotocin‐induced diabetic nephropathy in rats via hypoglycemic, antioxidant, anti‐inflammatory, and anti‐apoptotic effect involving the Nrf2 antioxidant pathway (Wahba et al. 2025). The Nrf2 pathway is therefore likely targeted coincidently by the sesquiterpene lactone and the S1R agonist to potent anti‐oxidant and anti‐inflammatory actions. Combination with the TSPO activator PK11195 was also largely synergistic but the effect may likely proceed from a different cellular mechanism. Indeed, PK11195 was shown to protect cognitive function in LPS‐injected mice by normalizing elevated inflammatory proteins expression, cyclooxygenase (COX)‐2, Aβ, with β‐site APP cleaving enzyme‐1 (BACE‐1) and insulin‐degrading enzyme (IDE) (Ma et al. 2016). In vitro, 4′‐chlorodiazepam, another TSPO ligand, was neuroprotective against Aβ_1‐42_‐induced toxicity in SH‐SY5Y neuroblastoma cells by increasing expression of SOD, thus by a different anti‐oxidant pathway (Arbo et al. 2017). In this latter combination, the combined activity on different anti‐oxidant and anti‐inflammatory pathways finally resulted in a synergic efficacy, with clear therapeutic potentialities.

It is crucial to assess the metabolite XPc in its intrinsic structure since it is made up of a sesquiterpene lactone, identified as the xanthatine, and amino acid proline added by Michael's additional reaction. This chemical reaction constitutes an intrinsic capacity of the sesquiterpene lactones, particularly when they are cytotoxic, and they react with thiols, such as free sulfhydryl group, mainly amino acid cystine in proteins (Tuasha et al. 2022). A specific trait of the XPc metabolite is its medium‐hydrophilic property, which is between the xanthatine hydrophobic and high‐hydrophilic proline traits. In concordance, the literature reported that the sesquiterpene lactones' activities can be affected by molecular geometry, lipophilicity, and chemical environment of the target sulfhydryl group (Tuasha et al. 2022). Xanthatine has been largely studied for its antitumoral effect, as attested by the publications (Takeda et al. 2022; Berenguer et al. 2024), which report the effective concentrations around 4 and 4.5 μg/mL (16.1 and 18.4 μM) and 20 μM, respectively. The same antitumoral effect has been described for the Onopordopicrin among those already described previously, which induced ROS accumulation and cell cytotoxicity at high concentrations (El Khatib et al. 2021). So, sesquiterpene lactones, such as onopordopicrin or xanthatine, can act as pro‐oxidants under high concentrations. In the work previously published, we tested XPc at a concentration of 90 μg/mL (24 μM) without cytotoxicity (Idres et al. 2021). We can hypothesize that adding proline to xanthatine could prevent cytotoxicity. To understand the XPc effects, the antioxidant mechanism involved, and the anti‐inflammatory property, it will be necessary to understand the role of the proline part of the XPc. The docking approach previously used with the G6PD tridimensional structure model (Idres et al. 2021) can be helpful to build some xanthatine derivatives by Michael's additional reaction to add a proline substitute molecule. A recent publication has evaluated new conjugates of serotonin with sesquiterpene lactones in the treatment of AD (Neganova et al. 2024).

## 5 Conclusion

We have demonstrated that XPc, an original sesquiterpene lactone derivative compound from burdock leaf, conjugate of proline with xanthatine has a preventive effect on AD development. Our experiments have shown the preventive of neuroinflammation and oxidative stress in the brain of Aβ_25‐35_‐treated mice. We have shown that XPc is a compound that protects glial cells against oxidative stress. It remains to confirm the molecular targets of XPc, particularly its potential role as a G6PD activator and its consequences on antioxidative cell pathways. At this end, the expression levels of many genes coding for antioxidant enzymes will be evaluated using a transcriptomic approach. It will be interesting to use the AD mice model to experiment XPc in a treatment anti AD.

## Author Contributions

**Charlyne Barry‐Simonnet:** investigation, data curation, formal analysis, writing, review and editing. **Lucie Crouzier:** investigation, data curation, formal analysis, writing, review and editing. **Tristan Moujellil‐Legagneur:** investigation. **Hamza Chaieb‐Errass:** investigation. **Yanis A. Idres:** conceptualization, writing, review and editing. **Karine Ferrare:** conceptualization, writing, review and editing. **Marc Rolland:** investigation. **Guillaume Cazals:** investigation. **Patrick Poucheret:** conceptualization, writing, review and editing. **Tangui Maurice:** formal analysis, visualization, writing, original draft, conceptualization, writing, review and editing, funding acquisition, project administration. **Didier Tousch:** conceptualization, writing, review and editing, funding acquisition, project administration. The authors declare that AI was not used for the preparation of this manuscript.

## Funding

This work was supported by Université de Montpellier, Soutien à la Recherche 2023 Accélérateur d'Innovation.

## Conflicts of Interest

The authors declare no conflicts of interest.

## Supporting information


**Table S1:** Calculation of combination index (CI): Spontaneous alternation data. The table presents the protection percentage for each dose of each drug calculated from the percentage of alternation shown in Figure 8A,E,I and for the combinations shown in Figure 9A,E; the estimated concentrations of each drug in the mix (C_x,Drug_) and the calculated combination index (CI).
**Table S2:** Calculation of combination index (CI): Passive avoidance data. The table presents the protection percentage for each dose of each drug calculated from the step‐through latency shown in Figure 8C,G,K and for the combinations shown in Figure 9C,G; the estimated concentrations of each drug in the mix (C_x,Drug_) and the calculated combination index (CI).
**Table S3:** Statistical analyses.

## Data Availability

The data that support the findings of this study are available on request from the corresponding author. The data are not publicly available due to privacy or ethical restrictions.
