# Supplementary material for: Xanthatin‐13‐(Pyrrolidine‐2‐Carboxylic Acid), a Sesquiterpene Lactone Isolated From Burdock Leaf, Attenuated Aβ25‐35 Toxicity and Memory Deficits in a Pharmacological Mouse Model of Alzheimer's Disease
Source: Phytother Res. 2026 Mar 7;40(5):2824–43. doi: 10.1002/ptr.70294 (PMC13144428; doi:10.1002/ptr.70294)
Supplement: Supplementary file 1 — Table S1: Calculation of combination index (CI): Spontaneous alternation data. The table presents the protection percentage for each dose of each drug calculated from the percentage of alternation shown in Figure 8A,E,I and for the combinations shown in Figure 9A,E; the estimated concentrations of each drug in the mix (Cx,Drug) and the calculated combination index (CI). Table S2: Calculation of combination index (CI): Passive avoidance data. The table presents the protection percentage for each dose of each drug calculated from the step‐through latency shown in Figure 8C,G,K and for the combinations shown in Figure 9C,G; the estimated concentrations of each drug in the mix (Cx,Drug) and the calculated combination index (CI). Table S3: Statistical analyses. [file PTR-40-2824-s001.docx]

**Supplementary Table 1.** Calculation of combination index (CI): Spontaneous alternation data. The table presents the protection percentage for each dose of each drug calculated from the percentage of alternation shown in Figure 8A,E,I and for the combinations shown in Figure 9A,E ; the estimated concentrations of each drug in the mix (C_x,Drug_) and the calculated combination index (**CI**).

*Treatment (mg/kg IP) PP (%) C_x,XPc_ C_x,PK11195 or PRE-084_* ***CI***

XPc (0) 0.0 ± 16.2

XPc (0.3) -22.2 ± 22.5

XPc (1) 36.9 ± 15.8

XPc (3) 100.5 ± 15.6 ^1,2^

PK11195 (0) 0.0 ± 19.3

PK11195 (0.03) 46.4 ± 29.4

PK11195 (0.1) 70.0 ± 19.1

PK11195 (0.3) 78.9 ± 27.1

PK11195 (1) 95.1 ± 23.7 ^3,4^

PRE-084 (0) 0.0 ± 9.0

PRE-084 (0.1) 13.4 ± 9.9

PRE-084 (0.3) 35.6 ± 9.5

PRE-084 (1) 68.4 ± 9.0

PRE-084 (3) 71.6 ± 12.9 ^1,2^

Linear regression

XPc (0.3) + PK11195 (0.03) 70.7 ± 16.0 2.15 ± 0.34 0.22 ± 0.05 **0.27 ± 0.05**

XPc (0.3) + PK11195 (0.1) 80.3 ± 16.0 2.44 ± 0.39 0.25 ± 0.06 **0.52 ± 0.10**

XPc (1) + PK11195 (0.03) 97.6 ± 19.0 2.97 ± 0.48 0.31 ± 0.07 **0.43 ± 0.09**

XPc (0.3) + PRE-084 (0.1) 68.6 ± 17.3 2.18 ± 0.33 0.94 ± 0.09 **0.24 ± 0.03**

XPc (0.3) + PRE-084 (0.3) 103.5 ± 14.9 3.29 ± 0.49 1.42 ± 0.14 **0.30 ± 0.04**

XPc (1) + PRE-084 (0.1) 87.0 ± 20.3 2.76 ± 0.41 1.19 ± 0.11 **0.45 ± 0.05**

log regression

XPc (0.3) + PK11195 (0.03) 70.7 ± 16.0 2.14 ± 0.34 0.15 ± 0.04 **0.33 ± 0.07**

XPc (0.3) + PK11195 (0.1) 80.3 ± 16.0 2.91 ± 0.47 0.32 ± 0.07 **0.42 ± 0.08**

XPc (1) + PK11195 (0.03) 97.6 ± 19.0 5.06 ± 0.81 1.14 ±0.27 **0.22 ± 0.04**

XPc (0.3) + PRE-084 (0.1) 68.6 ± 17.3 1.84 ± 0.28 1.89 ± 0.18 **0.22 ± 0.03**

XPc (0.3) + PRE-084 (0.3) 103.5 ± 14.9 3.85 ± 0.58 14.45 ± 1.39 **0.10 ± 0.01**

XPc (1) + PRE-084 (0.1) 87.0 ± 20.3 2.72 ± 0.41 5.54 ± 0.53 **0.39 ± 0.05**

Percent protection (PP) was calculated using 100% for V-treated animals and 0% for Aβ_25-35_-treated animals. C_x,Drug_ was calculated using the linear- or log-regressions from responses with the drug alone: ^1^ y = 32.881x, R^2^ = 0.9125 (lin), ^2^ y = 72.118x + 46.842, R^2^ = 0.7530 (log_10_); ^3^ y = 317.66x, R^2^ = 0.7671 (lin), ^4^ y = 30.996x + 95.847, R^2^ = 0.9697 (log_10_); ^5^ y = 73.09x, R^2^ = 0.9598 (lin), ^6^ y = 39.617x + 57.596, R^2^ = 0.9562 (log_10_). CI in bold shows synergy.

**Supplementary Table 2.** Calculation of combination index (CI): Passive avoidance data. The table presents the protection percentage for each dose of each drug calculated from the step-through latency shown in Figure 8C,G,K and for the combinations shown in Figure 9C,G ; the estimated concentrations of each drug in the mix (C_x,Drug_) and the calculated combination index (**CI**).

*Treatment (mg/kg IP) PP (%) C_x,XPc_ C_x,PK11195 or PRE-084_* ***CI***

XPc (0) 0.0 ± 10.3

XPc (0.3) 31.4 ± 18.1

XPc (1) 69.9 ± 13.9

XPc (3) 93.0 ± 17.4 ^1,2^

PK11195 (0) 0.0 ± 10.3

PK11195 (0.03) 58.5 ± 22.0

PK11195 (0.1) 43.9 ± 21.6

PK11195 (0.3) 62.2 ± 20.6

PK11195 (1) 21.6 ± 22.6 ^3,4^

PRE-084 (0) 0.0 ± 12.4

PRE-084 (0.1) 7.1 ± 9.2

PRE-084 (0.3) 24.6 ± 14.3

PRE-084 (1) 50.1 ± 13.8

PRE-084 (3) 18.4 ± 14.9 ^5,6^

Linear regression

XPc (0.3) + PK11195 (0.03) 66.9 ± 19.3 1.88 ± 0.28 0.27 ± 0.05 **0.27 ± 0.05**

XPc (0.3) + PK11195 (0.1) 43.8 ± 16.6 1.23 ± 0.18 0.18 ± 0.03 0.80 ± 0.14

XPc (1) + PK11195 (0.03) 30.8 ± 11.1 0.87 ± 0.13 0.13 ± 0.02 1.39 ± 0.24

XPc (0.3) + PRE-084 (0.1) 51.2 ± 18.9 1.17 ± 0.2 0.97 ± 0.13 **0.36 ± 0.06**

XPc (0.3) + PRE-084 (0.3) 86.0 ± 18.7 1.96 ± 0.34 1.63 ± 0.22 **0.34 ± 0.05**

XPc (1) + PRE-084 (0.1) 66.0 ± 21.2 1.50 ± 0.26 1.25 ± 0.17 **0.75 ± 0.12**

log regression

XPc (0.3) + PK11195 (0.03) 66.9 ± 19.3 1.09 ± 0.16 2.00 ± 0.39 **0.29 ± 0.05**

XPc (0.3) + PK11195 (0.1) 43.8 ± 16.6 0.47 ± 0.07 0.012 ± 0.002 9.01 ± 1.55

XPc (1) + PK11195 (0.03) 30.8 ± 11.1 0.29 ± 0.04 0.001 ± 0.000 49.05 ± 8.44

XPc (0.3) + PRE-084 (0.1) 51.2 ± 18.9 0.51 ± 0.09 1.47 ± 0.20 **0.65 ± 0.10**

XPc (0.3) + PRE-084 (0.3) 86.0 ± 18.7 1.37 ± 0.24 15.97 ± 2.19 **0.24 ± 0.04**

XPc (1) + PRE-084 (0.1) 66.0 ± 21.2 0.78 ± 0.14 4.04 ± 0.55 1.31 ± 0.20

Percent protection (PP) was calculated using 100% for V-treated animals and 0% for Aβ_25-35_-treated animals. C_x,Drug_ was calculated using the linear- or log-regressions from responses with the drug alone: ^1^ y = 35.521x, R^2^ = 0.8763 (lin), ^2^ y = 63.506x + 64.47, R^2^ = 0.6614 (log_10_); ^3^ y = 245.83x, R^2^ = 0.6614 (lin), ^4^ y = 10.362x + 63.728, R^2^ = 0.9253 (log_10_); ^5^ y = 52.903x, R^2^ = 0.97276 (lin), ^6^ y = 33.57x + 45.615, R^2^ = 0.9427 (log_10_). CI in bold shows synergy.

**Supplementary Table 3.** Statistical analyses.

***Figure*** *ANOVA Calculation Significance*

**1B** One-way *F*_(4,88)_ = 16.884, *p* < 0.0001 *******

**1C** One-way *F*_(4,88)_ = 2.499, *p* = 0.0483 *****

**1D** Kruskal-Wallis *H* = 19.17, *p* = 0.0007 *******

**1E** Kruskal-Wallis *H* = 11.43, *p* = 0.0221 *****

**2A** One-way *F*_(4,63)_ = 0.8531, *p* = 0.4971

**2B** One-way *F*_(4,63)_ = 0.3838, *p* = 0.8164

**2C** One-way *F*_(4,63)_ = 0.3073, *p* = 0.8720

**2D** One-way *F*_(4,63)_ = 0.4291, *p* = 0.7871

**2E** One-way *F*_(4,63)_ = 3.646, *p* = 0.0098 ******

**2F** One-way *F*_(4,63)_ = 4.072, *p* = 0.0053 ******

**3A** Repeated-measure one-way

Sc.Aβ/V *F*_(2.980,29.80)_ = 130.36, *p* < 0.0001 for swim ***

*F*_(10,40)_ = 2.427, *p* = 0.0229 for matching *

Aβ_25-35_/V *F*_(3.109,34.20)_ = 1.956, *p* = 0.1372 for swim

*F*_(11,44)_ = 4.293, *p* = 0.0002 for matching ***

**3B** Repeated-measure one-way

Aβ_25-35_/XPc (0.3) *F*_(3.273,29.46)_ = 4.224, *p* = 0.0115 for swim *

*F*_(9,36)_ = 4.054, *p* = 0.0012 for matching **

Aβ_25-35_/XPc (1) *F*_(3.446,37.90)_ = 8.477, *p* = 0.0001 for swim ***

*F*_(11,44)_ = 4.349, *p* = 0.0002 for matching ***

Aβ_25-35_/XPc (3) *F*_(3.118,34.29)_ = 6.578, *p* = 0.0011 for swim **

*F*_(11,44)_ = 4.753, *p* < 0.0001 for matching ***

**3C** One-way *F*_(4,50)_ = 3.000, *p* = 0.0268 *****

**3D** One-way *F*_(4,50)_ = 1.840, *p* = 0.1358

**3E** One-way *F*_(4,50)_ = 3.946, *p* = 0.0073 ******

**3F** Repeated-measure one-way

Sc.Aβ/V *F*_(2.052,20.52)_ = 12.27, *p* = 0.0003 for swim ***

*F*_(10,40)_ = 3.133, *p* = 0.0048 for matching **

Aβ_25-35_/V *F*_(2.920,32.12)_ = 11.08, *p* < 0.0001 for swim ***

*F*_(11,44)_ = 10.42, *p* < 0.0001 for matching ***

**3G** Repeated-measure one-way

Aβ_25-35_/XPc (0.3) *F*_(1.953,17.58)_ = 22.68, *p* = 0.0003 for swim ***

*F*_(9,36)_ = 7.506, *p* < 0.0001 for matching ***

Aβ_25-35_/XPc (1) *F*_(2.401,26.41)_ = 17.47, *p* < 0.0001 for swim ***

*F*_(11,44)_ = 3.066, *p* = 0.0040 for matching **

Aβ_25-35_/XPc (3) *F*_(2.362,23.62)_ = 13.36, *p* < 0.0001 for swim ***

*F*_(10,40)_ = 2.726, *p* = 0.0118 for matching *

**3H** One-way *F*_(4,50)_ = 0.8066, *p* = 0.5267

**4A** One-way *F*_(4,51)_ = 1.579, *p* = 0.1940

**4B** One-way *F*_(4,51)_ = 1.047, *p* = 0.392

**4C** One-way *F*_(4,51)_ = 1.677, *p* = 0.1697

**4D** One-way *F*_(4,51)_ = 1.244, *p* = 0.3043

**4F** One-way *F*_(4,23)_ = 2.868, *p* = 0.0460 *****

**5D** Two-way *F*_(1,16)_ = 1.241, *p* = 0.2817 for XPc

*F*_(1,16)_ = 73.55, *p* < 0.0001 for Aβ_25-35_ ***

*F*_(1,16)_ = 7.011, *p* = 0.0175 for the interaction *

**5F** Two-way *F*_(1,16)_ = 0.002218, *p* = 0.9630 for XPc

*F*_(1,16)_ = 4.756, *p* = 0.0445 for Aβ_25-35_ *

*F*_(1,16)_ = 1.578, *p* = 0.2270 for the interaction

**5G** Two-way *F*_(1,16)_ = 0.8642, *p* = 0.3664 for XPc

*F*_(1,16)_ = 2.271, *p* = 0.1513 for Aβ_25-35_

*F*_(1,16)_ = 4.949, *p* = 0.0408 for the interaction *

**6D** Two-way *F*_(1,16)_ = 29.17, *p* < 0.0001 for XPc ***

*F*_(1,16)_ = 25.72, *p* = 0.0001 for Aβ_25-35_ ***

*F*_(1,16)_ = 14.51, *p* = 0.0015 for the interaction **

**6F** Two-way *F*_(1,16)_ = 10.60, *p* = 0.0050 for XPc **

*F*_(1,16)_ = 4.514, *p* = 0.0495 for Aβ_25-35_ *

*F*_(1,16)_ = 17.48, *p* = 0.0007 for the interaction ***

**6G** Two-way *F*_(1,16)_ = 1.207, *p* = 0.2881 for XPc

*F*_(1,16)_ = 1.857, *p* = 0.1919 for Aβ_25-35_

*F*_(1,16)_ = 10.67, *p* = 0.00489 for the interaction **

**7A** One-way *F*_(4,29)_ = 4.317, *p* = 0.0073 ******

**7B** One-way *F*_(4,29)_ = 7.461, *p* < 0.0003 *******

**7C** One-way *F*_(4,37)_ = 16.65, *p* < 0.0001 *******

**7D** One-way *F*_(4,31)_ = 3.191, *p* = 0.0264 *****

**8A** One-way *F*_(4,61)_ = 11.88, *p* < 0.00001 *******

**8C** Kruskal-Wallis *H* = 25.30, *p* < 0.0001 *******

**8E** One-way *F*_(5,79)_ = 3.677, *p* = 0.0048 ******

**8G** Kruskal-Wallis *H* = 19.46, *p* = 0.0016 ******

**8I** One-way *F*_(5,183)_ = 15.81, *p* < 0.0001 *******

**8K** Kruskal-Wallis *H* = 30.52, *p* < 0.0001 *******

**9A** One-way *F*_(4,48)_ = 5.533, *p* = 0.0010 *******

**9C** Kruskal-Wallis *H* = 18.93, *p =* 0.0008 *******

**9E** One-way *F*_(4,65)_ = 10.84, *p* < 0.0001 *******

**9G** Kruskal-Wallis *H* = 17.43, *p* = 0.0016 ******
